# Race and Mortality Revisited

**DOI:** 10.1007/s12115-014-9790-1

**Published:** 2014-07-09

**Authors:** James P. Scanlan

**Affiliations:** Law Office of James P. Scanlan, 1529 Wisconsin Ave., NW, Washington, DC 20007 USA

In an article in the January/February 2000 issue of *Society* titled “Race and Mortality,” I explained the statistical pattern, inherent in other than highly irregular risk distributions, whereby the rarer an outcome, the greater tends to be the relative (percentage) difference between the rates at which advantaged and disadvantaged groups experience the outcome and the smaller tends to be the relative difference between rates at which such groups avoid the outcome. By way of example with respect to the health and healthcare outcomes on which the article principally focused, as mortality declines, relative differences in mortality tend to increase while relative differences in survival tend to decrease; as healthcare generally improves, relative differences in receipt of appropriate care tend to decrease while relative differences in failing to receive such care tend to increase. In 2000, however, this pattern was virtually unknown to health disparities researchers or anyone else examining demographic differences in favorable or adverse outcomes. Consequently, most efforts to appraise demographic differences in such outcomes were fundamentally unsound.

This article addresses the extent to which the appraisal of demographic differences in outcome rates is any sounder today than it was in 2000. In summary, while there has been increasing recognition of the ways that relative differences in outcome rates tend to be systematically affected by the prevalence (frequency) of an outcome, that recognition has yet to affect the way observers analyze group differences in outcome rates in any context. Though today vastly greater resources are devoted to the study of disparities in health and healthcare outcomes than in 2000, almost nothing said about such things as whether those disparities have increased or decreased over time or are otherwise larger in one setting than another, or even whether a disparity should be deemed large or small, has had a sound statistical basis. Meanwhile, federal regulators encourage mortgage lenders and public schools to reduce the frequency of adverse borrowing and student discipline outcomes in order to reduce the commonly observed severalfold racial and ethnic differences in rates of experiencing those outcomes. Neither the regulators, the congressional committees monitoring regulator policies, nor the institutions reducing the frequency of those outcomes in response to federal encouragements understand that reducing any outcome tends to increase, not reduce, relative differences in experiencing it. More broadly, events since 2000 do little to bolster one’s faith in the validity of accepted scholarship or the capability of individuals or institutions to recognize and acknowledge that things they have been doing for decades or generations have been incorrect or misleading.

## Relative Differences in Favorable and Adverse Outcomes

The pattern by which relative differences in experiencing and avoiding an outcome exhibit reverse correlations with the prevalence of an outcome can be well illustrated with hypothetical test score data. Suppose that at a particular cutoff failure rates are 20 % for an advantaged group (AG) and 37 % for a disadvantaged group (DG). At this cutoff DG’s failure rate is 1.85 times AG’s failure rate (37 %/20 %), while AG’s pass rates is 1.27 times DG’s pass rate (80 %/63 %).^1^ If the cutoff is lowered to a point where only 5 % of AG fails the test, assuming normal test score distributions, DG’s failure rate would be about 13 %. With the lower cutoff, DG’s failure rate would be 2.60 times AG’s failure rate (13 %/5 %), while AG’s pass rate would be only 1.09 times DG’s pass rates (95 %/87 %). Thus, when test failure became less common, the relative difference in failure rates increased while the relative difference in pass rates decreased.

The numbers in this example are set out in Table 1. The table also shows the absolute (percentage point) difference between the pass (and fail) rates of the two groups. But I will defer for some pages discussion of that measure and the way that it, too, tends to be systematically affected by the prevalence of an outcome.

**Table 1:** Fail and pass rates of advantaged group (AG) and disadvantaged group (DG) at different cutoffs, with measures of difference between rates

Cutoff	AG fail	DG fail	AG pass	DG pass	DG/AG fail ratio	AG/DG pass ratio	Percentage point diff
High	20 %	37 %	80 %	63 %	1.85	1.27	17
Low	5 %	13 %	95 %	87 %	2.60	1.09	8

A similar pattern can be found in virtually any data showing the proportions of groups defined by race, gender, education, income, or any other characteristic falling above or below different points on a continuum of quantifiable factors associated with experiencing an outcome or simply showing the rates at which the different groups experience or avoid an outcome at different levels of overall prevalence. Income data show that generally lowering poverty will tend to increase relative differences in poverty rates while reducing relative differences in rates of avoiding poverty, while general increases in poverty will have the opposite effect.

Table 2 illustrates these effects using data from my Spring 2006 *Chance* editorial titled “Can We Actually Measure Health Disparities?” The table presents the same type of information as in Table 1. But in this case the adverse outcome is having an income below 125 %, 100 %, or 75 % of the poverty line, while the favorable outcome is having an income above those points.

**Table 2:** Rates at which whites and blacks fall above and below 125 %, 100 %, and 75 % of the poverty line, with measures of differences between rates (2004)

Percent of poverty line	White percent below	Black percent below	White percent above	Black percent above	B/W below ratio	W/B above ratio	Percentage point diff	EES
125 %	14.9 %	31.0 %	85.1 %	69.0 %	2.08	1.23	16.1	0.54
100 %	10.8 %	24.7 %	89.2 %	75.3 %	2.29	1.18	13.9	0.55
75 %	7.2 %	17.8 %	92.8 %	82.2 %	2.48	1.13	10.6	0.54

The final column presents a figure identified as “EES” for “estimated effect size,” which is a measure of association unaffected by the prevalence of an outcome. But, as with the absolute difference, I will defer discussion of the measure for some pages.

For instant purposes, I merely note that the second and third rows of the table demonstrate the previously mentioned contrasting effects of decreases in poverty on relative differences in rates of experiencing and avoiding poverty, as where, for example, everyone previously with an income above 75 % of the poverty line is able to escape poverty (i.e., an increase in the relative difference between black and white poverty rates and a decrease in the relative difference between black and white rates of avoiding poverty). The second and first rows similarly illustrate the contrasting effects on the two relative differences where there occurs an increase in poverty such as to pull into poverty everyone previously with an income below 125 % of the poverty line (i.e., a decrease in the relative difference between black and white poverty rates and an increase in the relative difference between black and white rates of avoiding poverty).

The reader would be well served at this point to fully grasp the meaning of the two rate ratio columns in Table 2. And, in light of the implications of the patterns of changes in those columns as the table simulates general changes in the prevalence of poverty, the reader should consider whether there ever could be circumstances warranting the devoting of resources to exploring why black-white differences in either poverty rates or rates of avoiding poverty changed during periods of general increases or decreases in poverty without consideration of the patterns described here. That is, could it make sense, for example, to attempt to determine whether a particular administration’s manner of enforcing civil rights laws had a role in those changes without consideration of the extent to which the differences between rates changed simply because there occurred a general increase or decrease in poverty? If the answer to that question is not yet clear, it should become clear enough in due course.

National Health and Nutrition Survey data show that generally lowering blood pressure will tend to increase relative differences in hypertension while reducing relative differences in rates of avoiding hypertension and that generally improving folate levels will tend to increase relative differences in low folate while reducing relative differences in adequate folate; credit score data show that lowering a credit score requirement will tend to increase relative differences in failing to meet it while reducing relative differences in meeting it. Similarly, published life tables show that relative racial and gender differences in mortality are generally greater among the young than the old, while relative differences in survival are generally greater among the old than the young. Many tabular and graphical illustrations of the pattern by which the two relative differences tend to be affected by the prevalence of an outcome based on a wide range of data, including that from varied studies in medical and health policy journals, are available by means of the pages and subpages devoted to measurement issues on jpscanlan.com.^2^

Despite the fact that the described pattern by which the two relative differences tend to be affected by the prevalence of an outcome is evident in so many publicly available types of data, as well as hundreds or thousands of published studies – and that I had been describing it in various, sometimes prominent, forums since 1987^3^ – when “Race and Mortality” was published in 2000, the pattern was yet to be recognized in the wide range of activities in the law and the social and medical sciences where observers relied on relative differences in some favorable or adverse outcome to quantify demographic differences. Indeed, so far as the published record reflects, no one analyzing group differences recognized that it was possible for the two relative differences to change in opposite directions, much less that they tend to do so systematically. Following substantial declines in mortality and other adverse health outcomes in preceding decades, with corresponding increases in relative differences in experiencing such outcomes, observers took for granted that differences in the health of advantaged and disadvantaged groups had increased. Sometimes they even noted that the increases occurred “despite” general declines in the adverse outcome. But no one studying the issues either in the United States or abroad recognized the extent to which increasing relative differences in adverse health outcomes were to be expected simply because of the general declines in such outcomes and without consideration of whether relative differences in the opposite (favorable) outcomes had decreased.

Similarly, observers drew a range of inferences from the fact that relative racial or socioeconomic differences in adverse outcomes tended to be large among advantaged populations or subpopulations. Invariably, however, they failed to consider the reasons to expect large relative difference in adverse outcomes to be large wherever such outcomes are comparatively rare.

On the other hand, as rates of receipt of beneficial healthcare procedures like immunization and mammography increased, relative differences in receipt of such procedures tended to decrease while relative differences in failing to receive them tended to increase. And, because the convention was to measure healthcare disparities in terms of relative differences in favorable outcomes, disparities in such outcomes commonly were deemed to be decreasing. But here, too, such appraisals were made without regard to the extent to which the observed patterns were functions of general increases in receipt of such procedures.

As explained in “Race and Mortality,” the pattern by which the two relative differences tend to change in opposite direction as the prevalence of an outcome changes will not be found in every situation where one examines the sizes of relative differences at different points in time or in settings differentiated other than temporally. Observed patterns are also influenced by the comparative sizes of the differences between the risk distributions of advantaged and disadvantaged groups in the settings being examined. We might also characterize that factor as (a) the difference in the circumstance of the groups reflected by their outcome rates, (b) the strength of the forces causing the groups’ outcome rates to differ, or (c) the strength of the association between group membership and the outcome. The purpose of examining differing outcome rates of advantaged and disadvantaged groups is to understand this aspect of the matter in order, for example, to determine whether forces causing outcome rates to differ have increased or decreased over time and what factors contribute to such increases or decreases. But measures of differences between outcome rates that change solely because there occur overall changes in the prevalence of an outcome akin to that effected by lowering a test cutoff cannot provide useful information on such issues unless examined with an understanding of the way the measures tend to change solely because of changes in the prevalence of the outcome.

Many findings about directions of changes in the comparative status of two groups based on some standard measure might have been broadly correct because they were consistent with those one would reach while accounting for the ways measures tend to change as the prevalence of an outcome changes or were consistent with interpretations based on a measure unaffected by changes in prevalence of an outcome. Even then, however, such findings were misleading by implying that the employed measures could effectively quantify the differences in circumstances signified by the rates being examined. This was the case with respect to the interpretation of data on group differences in outcome rates with respect to every subject where the comparative circumstances of advantaged and disadvantaged groups was deemed a matter of consequence and regardless of the nature of the factors that caused those circumstances to differ.

Though “Race and Mortality” was not my first description of the ways the two relative differences tend to be affected by the prevalence of an outcome, it was the most comprehensive explication of the subject to date and appeared in a prestigious social science magazine. And, in addition to addressing many of the misunderstandings in the burgeoning field of health disparities research, the article touched upon a number of topical issues where observers commonly misinterpreted data because they failed to understand the ways the measures they employed tended to be affected by the prevalence of an outcome. It was also the first substantial articulation of those ideas in the Internet Age, when the widespread circulation of information was far easier than ever before. “Race and Mortality” thus had the potential to radically alter the way commentators and scholars, as well as law enforcement officials and courts, interpreted data on demographic differences in rates of experiencing some favorable or adverse outcome.

## Response of the National Center for Health Statistics to “Race and Mortality”

“Race and Mortality” highlighted the Race and Health Initiative, a federal program undertaken in 1998 with the intention to spend $400 million dollars over five years to address what were perceived to be the starkest racial disparities in health and healthcare. In subsequent years, the funds devoted to such research increased dramatically. In November 2000, Congress passed the Minority Health and Health Disparities Research and Education Act of 2000 establishing within the National Institutes of Health (NIH) the National Center on Minority Health and Health Disparities (NCMHD) (a center that would eventually be raised to the status of an Institute by the Patient Protection and Affordable Care Act of 2010). By 2003, almost 3 billion dollars yearly was being devoted to health and healthcare disparities research, or about nine percent of the NIH budget, while foundations and educational institutions, as well as state and local governments, also devoted increasing resources to such work. Since 2002, NCMHD has established more than eighty Centers of Excellence at universities and other institutions to develop ways to reduce health and healthcare disparities, and programs abound to train administrators and researchers in methods for monitoring disparities. Meanwhile, pursuant to legislation passed in 1999, commencing with fiscal year 2003, the Agency for Healthcare Research and Quality (AHRQ) has issued yearly *National Healthcare Disparities Reports* to document health and healthcare disparities and determine whether they are increasing or decreasing. In 2011 and 2013, the Centers for Control and Prevention (CDC) also issued extensive *Health Disparities and Inequalities Reports*.

Thus, many billions of dollars have been devoted to the study of health and healthcare disparities. But, while such research has effectively shown that demographic differences exist as to many outcomes, none of that research has considered the extent to which a particular measure used to appraise the size of a disparity tends to be affected by the prevalence of an outcome. Thus, efforts to determine whether such disparities have increased or decreased or what factors caused them to do so have rarely been of value and may at times have caused harm beyond the waste of resources and the misleading of the public and policy makers entailed in all unsound research.

That is not to say that measurement issues have been ignored. In “Race and Mortality” I alluded to an exchange in 1999 in which the Director of the National Center for Health Statistics (NCHS) suggested that, while NCHS had not previously considered the issues I raised, it would do so in the future. But NCHS statisticians actually responsible for developing the agency’s approach to the measurement of health and healthcare disparities were still unaware of those issues when I brought “Race and Mortality” to their attention in August 2001. They did, however, take considerable interest in the issues it raised, and between 2004 and 2009, authored five official or unofficial items in some manner attempting to address those issues (Keppel et al. 2004, 2005; Keppel and Pearcy 2005, 2006, 2009). The most important of these was a 2005 NCHS monograph titled “Methodological Issues in Measuring Health Disparities” (Keppel et al. 2005), authored by the principal NCHS disparities measurement experts and six other experts in the field.^4^

The monograph cited “Race and Mortality” to the effect that determinations as to the directions of changes over time will turn on whether one examines relative differences in favorable outcomes or relative differences in adverse outcomes. It illustrated the point by showing that determination of whether the disparity between Hispanic and non-Hispanic white women over 40 with respect to mammography increased or decreased between 1990 and 1998 would turn on whether one examined relative differences in the receipt of mammography or relative differences in non-receipt of mammography. The mammography figures cited in the document, along with the same measures shown in Tables 1 and 2 are set out in Table 3. But in order to simplify matters somewhat, in Table 3 and subsequent tables I present rates only for the outcome (favorable or adverse) typically reported, leaving the reader to infer the rate for the opposite outcome (i.e., the arithmetic difference between the shown rate and 100 %).

**Table 3:** White and Hispanic mammography rates in 1990 and 1998, with measures of difference

Year	White mamm rate	Hispanic mamm rate	W/H ratio mamm	H/W ratio no mamm	Percentage point diff	EES
1990	52.7 %	45.2 %	1.17	1.16	7.5	0.19
1998	68.0 %	60.2 %	1.13	1.24	7.8	0.21

But the NCHS monograph did not discuss the implications of the pattern by which the two relative differences tend to be affected by the prevalence of an outcome with respect to such pattern’s calling into question the utility of either measure for appraising the strength of the forces causing two rates to differ without consideration of the pattern. Rather, it merely recommended that henceforth, in order to promote consistency, all disparities (including both health and healthcare) should be measured in terms of relative differences in adverse outcomes.

Given that the pattern whereby the two relative differences tend to change in opposite directions as the prevalence of an outcome changes had been almost universally unknown among those analyzing demographic differences, it may not be so remarkable that an institution of NCHS’s statistical expertise would have failed to recognize such pattern until I brought it to the agency’s attention. But once the pattern was recognized by NCHS and those assisting it, that they could regard the matter to be satisfactorily addressed by choosing one relative difference over the other, and without questioning the basic validity of either relative difference for appraising differences in the circumstances of two groups reflected by a pair of outcome rates, suggests a basic misunderstanding of why one examines differences in outcome rates. Health and healthcare disparities research largely justifies itself on the basis that it seeks to understand the forces that cause outcome rates to differ in order to mitigate those forces. The forces that cause favorable outcome rates to differ are the same forces that cause the corresponding adverse outcome rates to differ. Thus, arbitrarily choosing a measure that says the forces have changed in one direction over one that says the they have changed in the opposite direction fits incongruously into a massive governmental undertaking to address something deemed to be a societal problem of great significance.

In any case, the perceptual consequences of the NCHS recommendation, which would underlie Health and Human Services’ appraisals of achievement of the health disparities reduction goals in Healthy People 2010, are potentially substantial. A great many healthcare disparities that might previously have been deemed to be decreasing would now be deemed to be increasing and further improvements in healthcare would tend to be associated with increasing healthcare disparities. Researchers at NCHS and elsewhere who had been pondering reasons why disparities as to particular types of healthcare had been decreasing now would have to ponder reasons why disparities in the same types of care had been increasing. That does not mean that a sound measure was replaced with a flawed measure, but merely that a flawed measure that tended to misleadingly indicate a pattern of decreasing healthcare disparities was replaced with a flawed measure that tended to misleadingly indicate a pattern of increasing healthcare disparities. At the same time, the flawed measure that tended to misleadingly show increasing disparities in adverse health outcomes like morbidity and mortality during periods of general improvements in health was left in place.

But NCHS’s treatment of the matter, either in the 2005 monograph or elsewhere, has done little to make it widely known that the two relative differences may change in opposite direction or even to make it clear that NCHS measures all disparities in terms or relative differences in adverse outcomes. Though Healthy People 2010 measured all disparities in terms of relative differences in adverse outcomes, it described many healthcare disparities in terms of favorable outcomes. Only those who read the Technical Appendix will recognize, for example, that the relative differences reported for a favorable outcome like immunization is actually the relative difference for the corresponding adverse outcome, and only those who read the references cited in the Technical Appendix will appreciate that what is reported as an increase in the relative difference for some favorable outcome may in fact involve a decrease in that relative difference. And only those who read the references in those references are likely to appreciate that neither relative difference is providing useful information about the nation’s progress in addressing differences in the health- and healthcare-related circumstances of advantaged and disadvantaged groups.^5^ Various articles or presentations by NCHS personnel have contributed to the failure of understanding by commonly discussing disparities measurement issues, including nuances of those issues, without mention even of the possibility for the two relative differences to change in opposite directions (Keppel 2007; Keppel et al. 2007; Klein and Huang 2010).

The Agency for Healthcare Research and Quality (AHRQ) which publishes the yearly National Healthcare Disparities Report (NHDR), has yet to show a recognition that it is possible for the two relative differences to change in opposite directions or that any measure of differences between outcome rates tends to be systematically affected by the prevalence of an outcome.^6^ The same may be said of the Institute of Medicine of the National Academy of Sciences, which provides occasional guidance for the NHDR and issues its own reports on progress in reducing healthcare disparities.

AHRQ also funds a great deal of health and healthcare disparities research. But it is virtually certain that AHRQ officials making funding decision, like those seeking the funding, do so without any understanding of the ways the measures to be employed in the research are likely to be affected by the prevalence of the outcomes at issue. Exemplary of the process is a $10 million AHRQ contract with the Institute for Medicine and Public Health of the Vanderbilt University Medical Center aimed at evaluating the effectiveness of quality improvement in reducing disparities in health and healthcare. The contract yielded a 475-page, peer reviewed report, issued in August 2012, that cites 4258 sources that were examined in fulfillment of the contract. But the report reflects no recognition whatever of the way the various measures employed in those studies may be affected by the prevalence of an outcome or even that it is possible that various measures could yield different conclusions as to directions of changes in disparities. In discussing findings of various studies, the report does not identify the measures that were used.^7^

The Centers for Disease Control and Prevention (CDC), of which NCHS is a part, conducts a substantial amount of healthcare disparities research, particularly with regard to immunization, even apart from the previously mentioned 2011 and 2013 *Health Inequalities and Disparities Reports*. The reports, like the many studies CDC researchers have conducted of immunization disparities, mainly rely on absolute differences between rates as a measure of disparities (a subject discussed below), though in some instances also showing relative differences in adverse or favorable outcomes. To my knowledge, no CDC document (save in the sense that NCHS is part of CDC) has ever indicated that it is possible for the two relative differences to change in opposite directions, that any measure tends to change solely because the prevalence of an outcome changes, or that there exist situations where NCHS would reach different conclusions about the direction of changes in disparities from those CDC would reach. In a 2013 videocast on healthcare disparities, a CDC official discussing what the agency regards as great progress in reducing childhood immunization disparities observed that once rates reached the 95 % level there was little room for disparities. Meanwhile, a NCHS study (Keppel 2007) found the largest black-white healthcare disparity to be in the failure to have an ongoing source of care where the black and white rates of having such a source were 93.5 % and 96.9 %. In other words, CDC can find healthcare disparities to be negligible in essentially the same circumstances where its arm NCHS finds disparities to be greatest.

Some private researchers have been measuring healthcare disparities in terms of relative differences in adverse outcomes, with or without reference to the NCHS recommendation, and with or without employing the approach of describing the matter in favorable terms while analyzing the matter in adverse terms. Notable is 2009 study in *Cancer Epidemiology Biomarkers and Prevention* by Harper et al. that examined mammography disparities over a period when mammography use had increased substantially. The abstract noted that “area-socioeconomic disparities in mammography use increased by 161 %.” But that figure actually was for the change in relative differences in non-receipt of mammography. As commonly happens during periods of substantial general increases in mammography (or anything else), relative differences in receipt of the procedure decreased substantially.^8^

The fact that the NCHS recommendation is not universally followed, and may not even be widely known, among disparities researchers is reflected in an award winning study appearing in *Pediatrics* in 2008. Relying on relative differences in vaccination rates as a measure of disparity, Morita et al. found that a school-entry Hepatitis B vaccination requirement that dramatically increased overall vaccination rates also dramatically reduced racial and ethnic vaccination disparities. As shown in Table 4, which presents figures from the Morita study on black and white fifth and ninth graders, NCHS would have found dramatically increased disparities (while those like CDC that rely on absolute differences would have found increasing disparities for fifth graders and decreasing disparities for ninth graders, in accordance with the pattern discussed below).

**Table 4:** White and black Hepatitis B vaccination rates in grades 5 and 9 before and after imposition of school-entry vaccination requirement, with measures of difference

Grade	Year	Program	White vac rate	Black vac rate	B/W ratio no vac	W/B ratio vac	Percentage point diff	EES
5	1996	Pre	8 %	3 %	1.05	2.67	5	0.47
5	1997	Post	46 %	33 %	1.24	1.39	13	0.34
9	1996	Pre	46 %	32 %	1.26	1.44	14	0.37
9	1997	Post	89 %	84 %	1.45	1.06	5	0.24

But rarely when an outcome increases from being very uncommon to being very common, as occurred in the case of the type of immunization that was the subject of the Morita study, will one fail to find a dramatic decrease in relative differences between rates of experiencing the outcome and a dramatic increase in rates of failing to experience the outcome. I have yet, however, to see any study of immunization disparities recognize the implications of whether one examines relative differences in receipt of immunization or non-receipt of immunization, even in studies that rely on both measures.^9^

And notwithstanding the NCHS treatment of the matter in 2005, rarely will one find recognition that there exist two relative differences or that it is even possible for them to yield different conclusions as to the directions of changes over time. Particularly in the discussion of racial disparities in cancer outcomes, studies commonly refer to relative differences in survival and mortality interchangeably, often stating that they are examining survival differences, while in fact examining mortality differences. Invariably, they do so without recognizing that as survival generally increases, relative differences in survival tend to decrease while relative differences in mortality tend to increase, or that more survivable cancers tend to show smaller relative differences in survival but larger relative differences in mortality than less survivable cancers. Indeed, it is likely that most reports about the comparative size of cancer survival disparities by type of cancer or age group are in fact reports about relative differences in mortality and involve situations where the comparative size of the relative differences in survival is the opposite of that reported.^10^

While my focus here is principally upon the situation in the United States, it warrants note that there is little reason to expect other countries to follow, or even know about, the NCHS recommendation to measure all healthcare disparities in terms of relative differences in adverse outcomes. Thus, healthcare researchers in other countries will commonly reach opposite conclusions about the comparative size of disparities from those NCHS would reach. While citing the NCHS 2005 monograph, a 2013 World Health Organization *Handbook on Health Inequality Monitoring* measures healthcare disparities in terms of relative differences in favorable outcomes. And, for example, it reaches starkly different conclusions about the comparative size of socioeconomic disparities in attendance of a skilled person at birth in different countries from those NCHS would reach on the basis of relative differences in the absence of such attendance.^11^

Many guides to the measurement of health and health disparities have been produced in the United States and abroad since 2000, including a popular University of Michigan online guide (Lynch and Harper 2005), guides from the National Cancer Institute (Harper and Lynch 2006, 2007), and a recent document from Harvard Medical School and Massachusetts General Hospital (Weissman et al. 2011), which will receive further attention below. But, apart from a 2005 guide issued by a Public Health Observatory in the United Kingdom (Carr-Hill and Chalmers-Dixon 2005),^12^ the NCHS responses to “Race and Mortality” is the only guide reflecting the least understanding of the ways measures tend to be affected by the prevalence of an outcome.

## Absolute Differences and the Value Judgment Fallacy

In 2000 absolute differences between rates did not seem to be used often enough in the measurement of health disparities or other demographic differences to warrant attention in “Race and Mortality,” which, in any case, focused on common misunderstandings of the two relative differences. Since that time, however, the use of absolute differences to appraise demographic differences has increased considerably, particularly in analyses of racial/ethnic and socioeconomic disparities in healthcare.

The absolute difference between rates – in the test score hypothetical, 17 percentage points before and 8 percentage points after the cutoff was lowered – is unaffected by whether one examines the favorable or the adverse outcome. Hence, such measure will yield only one conclusion as to the comparative size of disparities in different settings. But, as suggested earlier, for a measure to usefully quantify the difference in the circumstances of two groups reflected by a pair of outcome rates the measure must remain constant when there occurs a change in overall prevalence akin to that effected by the lowering of a test cutoff. And, like the two relative differences, absolute differences tend to be systematically affected by the overall prevalence of an outcome, though in a more complicated way than the two relative differences. Roughly, as uncommon outcomes (below 50 % for both groups) become more common, absolute differences between rates tend to increase; as common outcomes (above 50 % for both groups) become even more common, absolute differences tend to decrease. The prevalence-related direction of change is harder to predict when the outcome is neither common nor uncommon or changes from being uncommon to common (or vice-versa) during a period examined.^13^

As I discuss further in the section on pay-for-performance, in the main, observers relying on absolute differences have tended to do so without mention that a relative difference could (or in fact would in the particular circumstances examined) yield an opposite conclusion about the comparative size of a disparity from that yielded by the absolute difference. That holds even when the relative difference yielding a contrary conclusion is the measure typically employed in the circumstances. But in recent years there has been increasing recognition of the importance of presenting both relative and absolute differences in reporting on health and healthcare disparities (King et al. 2012; Welch et al. 2012). In circumstances where the particular relative difference the observer happens to be examining yields a different conclusion about the comparative size of two disparities from that yielded by the absolute difference, it has been argued that the contrasting conclusions are both valid in their way and that observers must make a value judgment in choosing between them (Lynch and Harper 2005; Harper et al. 2010). So far such discussions have entirely ignored the existence of a second relative difference. They have done so, even though, irrespective of the patterns I describe here, anytime it is noted that a relative difference and the absolute difference yield different conclusions about the comparative size of two disparities, the unmentioned relative difference necessarily will yield a conclusion that is the opposite of that yielded by the mentioned relative difference and the same as that yielded by the absolute difference.^14^

But consideration of the notion that two measures yielding opposite conclusions about such things as whether a disparity has increased or decreased over time could both be in some way valid, or that one must employ a value judgment to chose between them, provides a useful focal point for demonstrating a number of things about differing outcome rates of advantaged and disadvantaged groups and what we can learn from them.

Table 5 presents four situations that we might initially regard as the hiring patterns of four employers who hire for similar jobs from the same labor market and where we are required to rank the employers in descending order of the likelihood that they made biased hiring decisions or the degree of bias in those decisions. The principles I intend to elucidate, however, would apply equally in a range of circumstances where one might wish to compare the size of selection or rejection disparities, including with respect to changes in the disparities over time or differences in the sizes of the disparities as to different types of jobs or as to candidates of differing qualification levels. I will refer to this table again in subsequent discussion of such things as the misguided inclusion of healthcare disparity measures as performance elements in pay-for-performance programs.

**Table 5:** Hypothetical hire rates of advantaged and disadvantage groups, with measures of difference

Employer	AG hire rate	DG hire rate	AG/DG ratio hire	DG/AG ratio rejection	Percentage point diff	Odds ratio
A	20.0 %	9.0 %	2.22 (1)	1.14 (4)	11.0 (4)	2.53 (1)
B	40.0 %	22.6 %	1.77 (2)	1.29 (3)	17.4 (2)	2.28 (3)
C	70.0 %	51.0 %	1.37 (3)	1.63 (2)	19.0 (1)	2.24 (4)
D	80.0 %	63.4 %	1.26 (4)	1.83 (1)	16.6 (3)	2.31 (2)

The columns following the hire rates contain the same three measures of differences between selection or rejection rates used in the earlier tables, as well as the ratio of AG’s odds of selection to DG’s odds of selection.^15^ The parenthetical numbers reflect rankings of the comparative likelihood of bias (or degree of bias), from greatest to smallest, according to the particular measure.

Those who measure disparities in terms of relative differences in favorable outcomes (as would commonly be done in an employment discrimination case involving hiring or promotion) would rank the employers A,B,C,D. Those who measure disparities in terms of relative differences in adverse outcomes (as would commonly be done in a lending discrimination case or in an investigation of disparities in school discipline, and as might also be done in an employment discrimination case where the favorable outcome is retention and the adverse outcome is termination) would rank them D,C,B,A, the opposite of the first approach. Those who measure disparities in terms of absolute difference between rates (as has been done in studies of lending disparities by the Federal Reserve Board and as is increasingly done in studies of public school proficiency disparities and healthcare disparities) would rank them C,B,D,A. Those who measure disparities in terms of odds ratios (as often would be done by those attempting to adjust for differences in characteristics by means of logistic regression) would rank them A,D,B,C, the opposite of the ranking according to the absolute difference.

I suggest, however, that would be manifestly absurd to maintain that one employer is more likely to be biased than another as to selection while the other is more likely to be biased as to rejection. It would be likewise absurd to say that contrasting interpretations as to likelihood of bias based on either of the two relative differences and the absolute difference (or odds ratio) could all be sound or that determining which employers are the more likely to be biased involves a value judgment. Rather, there can exist only one reality as to the comparative likelihood of bias of the employers reflected in the data, even though that reality may be difficult to divine. The same holds for the above-mentioned alternative formulations of the hypothetical in a hiring context, as it would in any other context where bias might be involved in the allocation of favorable and adverse outcomes among different demographic groups.

That there can be only one reality may be most evident when something like biased decision-making is at issue. But it is no less the case when the questions of concern are whether the forces causing health or healthcare outcome rates of advantaged and disadvantaged groups to differ have increased or decreased over time and whether the policies or persons responsible for the changes should be regarded with approval or disapproval. For, as with selection and rejection, the forces causing rates for any favorable outcome to differ are the same forces causing rates for the corresponding adverse outcome to differ.

What then would be the soundest ranking of the employers with regard to the likelihood or degree of bias in its hiring decisions? Each row of information is based on the specifications underlying the test score hypothetical at the outset – that is, normal risk distributions with means that differ by half a standard deviation. There thus is no rational basis for asserting that the strength of the forces causing the observed differences in hire (or rejection) rates to differ varies among any of the four situations reflected in the table, and any measure that suggests the strength of those forces does vary from situation to situation is a flawed measure.

## A Theoretically Sound Measure of the Forces Reflected by a Pair of Outcome Rates

Implicit in the illustration in Table 5 is that the only theoretically sound way to appraise the strength of the forces causing favorable or adverse outcome rates of advantaged and disadvantaged groups to differ is to derive from pairs of outcome rates the difference between the means of the underlying distributions of factors associated with experiencing the outcome at issue. I have commonly termed the figure so derived the estimated effect size (EES).^16^ The selection or failure rates in Table 5 by definition would yield an EES of 0.5 standard deviations, which would mean that approximately 31 % of the disadvantaged group’s distribution is above the mean for the advantaged group.^17^

This approach to appraising the strength of the forces causing outcome rates to differ is inexact in a number of respects. For example, it relies on an assumption that the underlying distributions of factors associated with experiencing an outcome are normal. Rarely can we be sure that the underlying distributions are normal and sometimes we will know that they are not normal, as, for example, when the distributions are truncated part of normal distributions. There also exists a range of more subtle issues.^18^ But an approach of this nature (including any that might be better informed as to actual shapes of the underlying risk distributions) is plainly superior to reliance on standard measures of differences between outcome rates without consideration of the ways the measures tend to be affected by the prevalence of the outcome at issue. For it provides a benchmark for appraising the strength of the association reflected by any pair of rates and for comparing the strengths of association reflected by two or more pairs of rates when standard measures would yield varying interpretations as to the comparative size of the differences between rates. And it can at least spare us from wrongly concluding, on the basis of one preferred standard measure or another, that there is reason to distinguish among the employers in Table 5 and then mistakenly devoting resources to exploring the reasons for the perceived differences, drawing inference based on the perceived differences, or making decisions of consequence based on the perceived differences.

The EES figures in Tables 2 through 4 provide us some perspective on the measure, with respect to both how we might regard the size of the particular disparity and what conclusions we might draw about changes over time. Table 2 indicates that differences in susceptibility to poverty are slightly larger than the above-described differences reflected by the hypothetical test score data. Given that the table reflects the consequences solely of changes in prevalence of poverty, that the EES figures change at all from row to row indicates an imperfection in the method in consequence of minor irregularities in the two distributions. But that the differences are minimal relative to in the size of the EES figures reflects the utility of the measure for estimating whether during times of changes in overall poverty, there occurred any meaningful change in the differences between the circumstances of advantaged and disadvantaged groups pertinent to likelihood of being in or out of poverty.

The EES figures in Table 3 for the Hispanic-white disparity in mammography suggest a rather smaller disparity than observed for the black-white difference in susceptibility to poverty. And while the EES increased slightly over the course of the period examined, the size of the change was such as to offer little reason to believe anything meaningful occurred regarding the strength of the forces causing Hispanic and white mammography rates to differ.

The EES figures in Table 4 suggest that the differences in black-white likelihood of Hepatitis B immunization, both before and after imposition of the vaccination requirement, are somewhere between the difference in black-white susceptibility to poverty reflected in Table 2 and the difference in Hispanic-white mammography reflected in Table 3. But the figures also indicate that the imposition of the vaccination requirement in fact caused the strength of the forces causing rates to differ (differences in the circumstances of the two groups) to noticeably decrease. That is something that it would seem reasonable to expect when a school-entry requirement is imposed. A rigidly enforced requirement should entirely eliminate any disparity. Further perspective on the EES figure is provided in tables discussed below.

## Absolute Differences and Pay-for-Performance

Most adverse health outcomes are in ranges where reductions in those outcomes tend to reduce absolute differences between rates of advantaged and disadvantaged groups. Were researchers to employ absolute differences to appraise things like racial differences in infant mortality, the common perception that demographic differences in such outcomes have been increasing would change to a perception that they have been decreasing. Recent reliance on absolute differences as a measure of healthcare disparity involves outcomes that can be in ranges where improvements will tend either to increase or reduce absolute differences. An illustration of the prevalence-related patterns may be found in Table 5, if one considers Rows A and B to reflect the before and after situations for an increase in an uncommon healthcare outcome and Rows C and D to reflect the before and after situations for an increase in a common healthcare outcome. The standard pattern of declining relative differences for the increasing outcome and increasing relative differences for the corresponding decreasing outcome exists in both situations. But the absolute difference increases in the former situation and decreases in the latter.

An instructive example of the failure to understand the ways absolute differences tend to be affected by the prevalence of an outcome may be found in two studies appearing in the same 2005 issue of the *New England Journal of Medicine*. Jha et al. relied on absolute differences between rates in examining racial disparities in rates of receiving certain fairly uncommon procedures where rates were generally increasing; and, as commonly happens when outcome rates in the ranges at issue are generally increasing, the absolute differences between rates usually increased. Trivedi et al. relied on absolute differences between rates in examining racial disparities in adequacy of care (including both treatment and control of conditions) where adequacy of care rates (especially as to treatment) were at generally high levels and increasing; and, as commonly happens in such circumstances, absolute difference between rates usually decreased (especially for treatment).

But neither study, nor a commentary discussing the contrasting findings (Lurie 2005), recognized that absolute differences tend generally to behave in the manner observed in each study irrespective of any changes in the forces causing rates of racial groups to differ. The same situation holds for all other efforts to date that have relied on absolute differences to determine whether healthcare disparities have increased or decreased over time or are larger in one setting than another (e.g., managed care versus fee-for-service care, as in Schneider et al. 2001) or with respect to one type of outcome compared with another (e.g., treatment of conditions versus control of conditions, as in Trivedi et al. 2006).

All research that relies on some measure without consideration of the way it is affected by the prevalence of an outcome wastes resources and misleads those who rely on it. But it is with respect to reliance on absolute differences in healthcare outcome rates in the pay-for-performance (P4P) context that the failure to understand the ways measures tend to be affected by the prevalence of an outcome may have the most concrete adverse consequences.

A 2005 study in *Circulation* by Werner et al. relied on absolute differences between rates in finding that a coronary artery bypass graft (CABG) report card program, which was believed to generally increase CABG rates, increased racial differences in such rates. The white rate had risen from 3.6 % to 8.0 % while the black rate had risen from 0.9 % to 3.0 %, with a resulting increase in the absolute difference from 2.7 percentage points to 5.0 percentage points. As discussed above, such rates are in ranges where general increases would commonly increase absolute differences without regard to any change in the strength of the forces causing rates of advantaged and disadvantaged group to differ.

These figures are set out in Table 6, along with the rate ratios for receipt and non-receipt of CABG and the EES. In addition to showing the increase in the absolute difference for this uncommon outcome, the table shows that the relative difference in receipt of the procedure decreased, while the relative difference in failure to receive the procedure (the NCHS approach) increased. In other words, each measure behaved in accordance with the prevalence-related patterns described above. The EES suggests that they did so even though, to the extent that the forces causing white and black rates to differ can be measured, such forces decreased.

**Table 6:** CABG rates of white and blacks before and after use of CABG report card, with measures of difference

Period	White CABG rate	Black CABG rate	W/B ratio CABG	B/W ratio no CABG	Percentage point diff	EES
Before	3.6 %	0.90 %	4.00	1.03	2.7	0.57
After	8.0 %	3.00 %	2.67	1.05	5.0	0.48

But without consideration of the prevalence-related patterns – or the fact there had occurred a decrease in the relative difference in the favorable outcome, which was probably the most common measure of such disparities at the time – the authors interpreted the increase in absolute differences to indicate that incentive programs would tend to increase healthcare disparities. Observers then uncritically employed the same reasoning to conclude from the study that the P4P programs being implemented across the county would tend to increase racial disparities in healthcare. In order to counter that tendency, they recommended that P4P programs include criteria for evaluating provider performance on the basis of the size of, or changes in the size of, healthcare disparities. Massachusetts responded to that recommendation by including a healthcare disparities criterion in its Medicaid P4P program, and it employed a measure of disparity that is a function of the absolute difference.

Any P4P program tying performance to some measure of disparity without consideration of the way the measure tends to be affected by the overall prevalence of an outcome would involve allocation of monetary incentives for reasons unrelated to the comparative equity at monitored institutions. And that would hold regardless of the measure used.

In cases where programs measure disparities in terms of changes in absolute differences over time, as noted several paragraphs above, improvements in healthcare for uncommon outcomes will tend to be perceived as increasing disparities, while improvements for common outcomes will tend to be perceived as reducing disparities. In rate comparisons across hospitals, higher-performing hospitals will tend to show larger disparities than lower-performing hospitals for uncommon outcomes but smaller disparities for common outcomes (as again reflected in Table 5 where rows A and B represent the lower-performing and higher-performing hospitals as to uncommon outcomes and Rows C and D represent such hospitals as to common outcomes). The Massachusetts program appraised disparities across hospitals and did so with respect to meeting some recommended standard of care where rates averaged above 80 % for all groups. Given the tendency for higher overall rates in such ranges to be associated with smaller absolute differences, the program will tend to find healthcare disparities to be smaller at higher-performing hospitals than lower-performing hospitals. It thus will tend to reward higher-performing hospitals for reasons unrelated to a useful indicator of cross-hospital equity. Further, since higher-performing hospitals tend to have smaller minority representations among their patient populations than lower-performing hospitals, the inclusion of a disparities criterion in the Massachusetts P4P program, by diverting resources away from providers with large numbers of minority patients, is more likely to increase healthcare disparities than to reduce them.^19^

Meanwhile, in the United Kingdom, reliance on absolute differences to measure healthcare disparities with respect to a fairly common outcome, where improvements tend to reduce absolute differences, has led to the perception in that country that P4P will tend to reduce healthcare disparities. UK researchers tend also to rely on absolute differences between rates to measure socioeconomic differences in cancer outcomes. Thus, in contrast to the research in the United States, which, as discussed, measures cancer outcome disparities in terms or relative differences in mortality (while terming them relative differences in survival) and tends to find increases in survival to be associated with increasing racial disparities, research in the UK tends to find general improvements in survival to be associated with increasing disparities for less survivable cancers and decreasing disparities for more survivable cancers.

## Illogical Expectations and Unfounded Inferences

In “Race and Mortality” I explained that implicit in the described pattern by which the two relative differences are affected by the prevalence of an outcome is a pattern whereby, when an outcome changes in prevalence, the group with the lower baseline rate will tend to experience a larger proportionate change in its rate of experiencing the outcome while the other group will tend to experience a larger proportionate change in its rate of experiencing the opposite outcome. For example, the hypothetical lowering of a test cutoff shown in Table 1 caused the failure rate of AG to decrease by 75 % compared with a 65 % reduction for DG, while causing the pass rate of DG to increase by 38 % compared with a 19 % increase for AG.

Yet it is commonly assumed that whenever something causes outcome rates to change it is somehow normal for different baseline rates to change the same proportionate amount and that something significant must have occurred whenever those rates are found to change by different proportionate amounts. Irrespective of the statistical pattern described above, however, the expectation of equal proportionate changes is illogical. For a factor cannot cause equal proportionate changes in two different baseline rates for experiencing some outcome while causing equal proportionate changes in the opposite outcome rates. That is, if a factor were to cause baseline rates of 20 % and 10 % to change equal proportionate amounts (say, doubling them to 40 % and 20 %), it will necessarily cause the opposite outcome rates to change different proportionate amounts (80 % reduced to 60 %, a 25 % reduction; 90 % reduced to 80 %, an 11 % reduction). Since there is no more reason to expect equal proportionate changes in one outcome than there is to expect equal proportionate changes in the opposite outcome, there is no reason to regard it as somehow normal to find equal proportionate changes in either outcome.

Nevertheless, when advances in healthcare cause larger proportionate reductions in adverse health outcomes among advantaged groups than disadvantaged groups, observers devise seemingly sophisticated theories to explain those patterns, such as the “diffusion of innovation” or the ‘inverse equity” hypotheses. But they devise these theories without consideration that the disadvantaged group has experienced the larger proportionate increase in the favorable outcome.

Similarly, in an increasingly common area of study called “reporting heterogeneity” observers find significance, for example, in the fact that a chronic health condition causes a larger proportionate increase in the reporting of less-than-good health among advantaged groups than disadvantaged groups and theories are posited as to what such pattern signifies. But the same studies would commonly show that such conditions reduce rates of good-or-better health proportionately more among disadvantaged than advantaged groups.

For as long as demographic differences in poverty have been studied observers have been reporting as if it were significant that poverty has increased or decreased proportionately more among advantaged groups than disadvantaged groups. But, as suggested by Table 2, when there occurs any substantial change in poverty, rarely will one fail to find that the poverty rates of groups with lower baseline rates changed proportionately more than other groups or that the other groups experienced larger proportionate changes in rates of avoiding poverty. That female-headed families experiencing a smaller proportionate decline in poverty than other groups during the substantial reductions in poverty between 1959 and the early 1970s was an important element in perceptions about the “feminization of poverty” – a misguided concept given several paragraphs in “Race and Mortality,” and referenced in note 3 above, but one as vibrant and misunderstood in 2014 as in 2000. Lately, however, researchers have been discussing the comparative size of percentage point changes in poverty, an approach that would have found female-headed families to have especially benefited from reductions in poverty half a century ago. Whatever the measure employed, however, poverty researchers have yet to address the extent to which observed patterns are functions of overall changes in poverty.

In clinical trials substantial resources are devoted to determining whether an intervention will cause different proportionate changes in the baseline rates of different subgroups (a phenomenon termed “subgroup effects,” “effect heterogeneity,” “interaction,” etc.) and there occurs frequent discussion about methods for determining whether observed differences in proportionate changes might merely reflect sampling variability rather than differences that exist in the population at large. Institutions providing guidance on evidence-based medicine commonly recommend that, absent sound evidence of a subgroup effect, as defined above, treatment decisions shall be based on the assumption that, for example, a factor that is observed to reduce a baseline rate of 20 % to 10 % in a clinical trial will cause a like 50 % reduction of all other baseline rates. Few question the soundness of this assumption.^20^

In “Race and Mortality” I discussed a number of situations where observers drew inferences based on the size of relative differences in a favorable or adverse outcome in some subpopulation without consideration of the role of the comparatively high or low prevalence of the outcome within the subpopulation. Usually these involved attention to a seemingly large relative difference in some adverse outcome within an advantaged subpopulation where the outcome tended to be rare, such as comparatively large racial differences in infant mortality where parents were highly-educated or comparatively large racial differences in rejection rates among high-income mortgage loan applicants. Another prominent example may be found in interpretations of occupational differences in the health of British civil servants in what are known as the Whitehall Studies. Such studies have found larger relative differences in adverse health outcomes in this relatively advantaged subpopulation, few of whose members suffered any material deprivation, than in the general UK population. The steeper gradient among civil servants than the general population has been interpreted as suggesting that differences in psychosocial factors and stresses arising from the workplace hierarchy are as important to health disparities as differences in material well-being, or that observed relative differences in adverse outcomes among the general population are smaller than would be observed if there existed indicators of socioeconomic status for the general population that are as precise as the occupational categories at Whitehall. Those drawing such inferences, however, have failed to consider that large relative differences in adverse outcomes are to be expected among British civil servants simply because they are a relatively healthy subpopulation or that relative differences in favorable outcomes are likely to be smaller among civil servants than the general UK population.

Similarly, researchers have noted the diminishing relative differences in adverse health outcomes among Whitehall retirees, opining that the removal from the stresses of a hierarchical working environment are the reason for the reduction in the disparity (Chandola et al. 2007). But, as in the common situation where one observes smaller relative differences in mortality (though larger relative differences in survival) among the old than the young, there is no basis for drawing any inferences based on a comparison of relative differences (as to either outcome) without consideration of the effects of prevalence on the chosen relative difference.^21^

Inferences based on the comparative size of relative differences in favorable outcomes are no less problematic than those based on the comparative size of relative differences in adverse outcomes. In the employment context, some observers would read smaller relative differences in selection rates among more credentialed applicants, where selections rates tend to be high (as in Rows C and D of Table 4), than among less-credentialed applicants, where selection rates tend to be low (as in Rows A and B), as indicating that employers are less inclined to rely on stereotypes when there exist objective indicators of qualifications. They draw that inference, however, unaware or ignoring that examination of relative differences in rejection rates would support an opposite inference. The mistaken interpretation of comparatively larger racial differences in mortgage rejection rates among high-income applicants discussed in “Race and Mortality” could just as well have been couched in terms that having higher income does not reduce chances of rejection as much for blacks as whites. But one study (Kim and Squires 1995) instead examined the effects of having higher income on mortgage approval rates of blacks and whites, finding that the increase in approval rates was greater for blacks than whites, and posited an explanation for that pattern. Whether or not the explanation was plausible, it lacked a statistical foundation.

Focusing on the negative factor of having a criminal record on the favorable outcome of receiving callback after a job interview for tester pairs comprised of two black or two white job applicants, a study (Pager 2003) finding that having a criminal record reduced callback rates proportionately more for blacks than whites posited an explanation for that pattern. Overlooked, however, was that the criminal record increased the rates of failure to receive a callback more for whites than blacks. The latter difference, which is the one NCHS would examine, would have required a different explanation. Data from the study are presented in Table 7.

**Table 7:** White and black rates of receiving callbacks for tester applicants with and without convictions indicated on their applications, with measures of difference

Race	No conviction callback rate	Conviction callback rate	No conv/Conv ratio callback	Conv/No conv ratio no callback	Percentage point diff	EES
White	34 %	17 %	2.00	1.26	17	0.54
Black	14 %	5 %	2.80	1.10	9	0.56

In this case, however, the measures of difference show the effect of a factor on blacks and whites rather than the effect of race. The table shows that the standard measures all behave in the way the prevalence-related forces typically would drive them. And the EES, which is also suitable for this purpose, indicates that the effect of having a criminal record was essentially the same for blacks and whites.^22^

An interesting thing about a study of this nature (also pertinent to any study where overall favorable outcome rates can be very low or very high) is that if the job market or the fabricated qualification of the tester pairs were such as to make chances of callback very high, there would be a tendency to examine relative effects on the uncommon outcome of failure to receive a callback. In such circumstances, the study would then have tended to find a greater effect of a criminal record on the failure to receive callbacks on whites than blacks, just as having low income would be found to increase mortgage rejection rates more for whites than for blacks.

More generally, in most cases where an observer draws an inference based on, or posits an explanation for, the comparative size of relative differences in a favorable or adverse outcome, the relative difference as to the opposite outcome would support a different inference or require a different explanation.^23^ But invariably the perception about the comparative size of the relative difference will lack a sound statistical basis. That holds as well with respect to inferences based on the comparative size of absolute differences.

## Lending and Discipline Disparities

The failure to understand the contrasting patterns by which relative differences in favorable outcomes are affected by the prevalence of an outcome is implicated in two perverse federal law enforcements policies that have been much in the news in recent years. In “Race and Mortality” I discussed the fact that out of concern that standard lending criteria were responsible for large racial differences in mortgage rejection rates federal regulators had encouraged lenders to relax those criteria. That approach accorded with longstanding policy concerning the racial impact of employment tests, where lowering cutoffs was regarded as a means of reducing such impact because it reduced relative difference in pass rates. Lenders who relaxed lending criteria in response to regulator encouragements presumably reduced relative differences in mortgage approval rates. But as shown at the outset, while lowering standards tends to reduce relative differences in satisfying them, it tends to increase relative differences in failing to satisfy them. Unaware of the latter pattern, regulators continued to monitor the fairness of lender practices on the basis of relative differences in rejection rates. Thus, by responding to federal encouragement to relax criteria lenders increased the chances that they would be sued for discrimination.

More recently the fair lending focus has been on differences between rates at which minorities and whites received subprime rather than prime mortgage loans, as in the suits underlying the $335 million and $175 million settlements against Countrywide Financial Corporation and Wells Fargo Bank that received widespread media attention in 2011 and 2012. But the complaints in both cases fault the lenders for policies that fail to minimize the proportion of loans that are subprime rather than prime, thus encouraging lenders to reduce the frequency of subprime loans. Because regulators continue to monitor the fairness of practices on the basis of relative differences in adverse outcomes, here, too, responsive lenders increase their risk of litigation.

For some years, the Departments of Education (DOE) and Justice (DOJ) have been attributing large relative racial and ethnic differences in suspension and expulsion rates to zero tolerance policies in effect in recent decades and have been encouraging schools to relax standards in order to reduce those differences. The agencies’ January 2014 release of school discipline guidance, with a joint focus on generally reducing discipline rates and reducing racial differences in discipline rates, is the most recent and most prominent reflection of the agencies’ views on the matter. Various jurisdictions have been relaxing standards while believing, in accordance with the government’s expressed views, that doing so will tend to reduce racial differences. Yet, as with any outcome, reducing the frequency of suspensions and expulsions will tend to increase, rather than reduce, relative differences in experiencing such outcomes. Indeed, a November 2012 DOE school equity report shows smaller relative racial differences in expulsions in districts with zero tolerance policies than in districts without such policies, and reductions in suspension rates in the states of California and Maryland and the cities of Los Angeles and Denver have been accompanied by increased relative racial/ethnic differences in suspensions.^24^ Meanwhile, the government continues to appraise the fairness of discipline policies on the basis of relative differences in those outcomes. As in the lending context, school districts responding to government encouragements to relax standards tend to increase the chances that the government will sue them for discrimination.

In March 2014, DOE and DOJ jointly released a discipline disparities report that included information on demographic disparities in suspensions from preschool programs. The report elicited great concern that preschool administrators would find reason to suspend preschoolers in other than the rarest of cases as well as concern over what were perceived to be huge racial disparities in suspension rates among preschoolers. No one grasped the connection between the two issues, specifically, that relative differences in suspension rates tended to be especially larger among preschoolers precisely because suspension are so rare in preschools.

Table 8 presents the figures from the report on multiple suspensions (which were the focus of much of the media coverage) both for preschool and K-12. Once again we observe the patterns that persons with an understanding of risk distributions would tend to expect – i.e., larger relative differences in the adverse outcome, but smaller relative differences in the corresponding favorable outcome, in the setting where the adverse outcome is less common. And the EES tells us that, whatever the forces causing multiple suspension rates of blacks and whites to differ, the strength of the forces is essentially the same in preschool as in K-12. Perceptions about the size of the preschool disparities, however, are likely to cause general reduction in suspension rates among preschoolers. That will tend to increase the relative differences prompting the reductions.

**Table 8:** White and black rates of multiple suspensions in preschool and K-12, with measure of difference

Level	White mult susp rate	Black mult susp rate	B/W ratio mult susp	W/B ratio no mult susp	Percentage point diff	EES
Preschool	0.15 %	0.67 %	4.41	1.01	0.52	0.49
K-12	2.23 %	6.72 %	3.01	1.05	4.49	0.51

The March 2014 report also found very large relative differences between the discipline rates of students with and without disabilities, something that had been noted in many previous studies. These differences have been a subject of sufficient concern that in the Individuals with Disabilities Education Improvement Act of 2004 Congress mandated that recipients of federal assistance with “significant discrepancies” in rates of long-term suspensions of disabled and non-disabled students must consider approaches to discipline of the type that commonly reduce suspension rates. Such discrepancies are invariably measured in terms of relative differences in adverse outcomes, which will tend to be greater where suspension rates are generally lower. Thus, the statute is likely to cause jurisdictions already with low suspension rates to further lower those rates, thus increasing the relative differences in suspensions that prompted the modifications.

The measurement issues discussed here are also implicated in interpretations about racial differences that DOE monitors regarding academic outcomes. But neither DOE nor others examining demographic differences in proficiency or non-proficiency and graduation or dropout rates recognize that overall changes in outcome rates commonly lead to an increase in the relative difference as to one outcome and a decrease in the relative difference as to the other outcome. Increasingly, proficiency disparities tend to be monitored in terms of absolute differences. But such monitoring is conducted without recognizing, for example, that overall improvements will tend to increase absolute differences for subjects with generally low proficiency rates but reduce absolute differences for subjects with generally high proficiency rates.^25^

## Looking Forward

In maintaining that all research into demographic differences in outcome rates without consideration of the way the measure employed is affected by the prevalence of an outcome has been fundamentally flawed or misleading, I proceed from the perspective that there is not much here about which reasonable people can differ. While few people are aware of the patterns reflected in Tables 1 and 2 or like patterns that can be illustrated with myriad other types of data, that does not make the existence of the patterns in any sense debatable. And once the patterns are recognized, one can hardly question their essential implications respecting the use of standard measures to quantify differences in the circumstances of two groups reflected by a pair of outcome rates. For example, to return to the question posed with regard to the patterns in Table 2, once one understands the patterns, there exists no plausible justification for exploring such things as the way particular policies may affect racial differences in susceptibility to poverty without consideration of those patterns.

That one may observe departure from these patterns does not alter the situation. Departures from the patterns in fact are the principal, if not only, things worth exploring. Nor does the accuracy of my descriptions of the patterns by which measure tend to change as the prevalence of an outcome change matter very much. As long as a measure tends to be in *any* way affected by the prevalence of outcome, one cannot reasonably rely on the measure to quantify the strength of an association without considering the implications of such effect. At any rate, no one has yet advanced a plausible rationale for doing so.

The matter is equally clear in the case of the federal fair lending and public school discipline contexts. Once recognizing that lowering a test cutoff tends to increase relative differences in failure rates, a thoughtful person cannot long fail to recognize that lowering a credit score requirement or increasing the number of classroom infractions necessary to merit a suspension will have like effect on relative differences in the pertinent adverse outcomes.

Since 2004, more often in Europe than the United States, researchers have been responding to “Race or Mortality” or some prior or subsequent articulation of its main points (including the 2006 *Chance* editorial). These responses are summarized in the Consensus subpage of the Scanlan’s Rule page of jpscanlan.com. While the responses do not necessarily recognize the way the described patterns are inherent features of the underlying risk distributions, they do recognize that measures will commonly show the correlations with overall prevalence that I have described. Such recognition, however, has failed to affect research practices, even among authors of works reflecting such recognition, and some of those authors have gone on to do further work while ignoring the implications of their own conclusions that measures employed in such work can only be useful if appraised with regard to the prevalence of the outcome studied.

In recent years, I have formally contacted institutions whose activities involve the interpretation of data on group differences advising them of the ways those activities are undermined by failure to recognize that standard measures of differences between outcome rates tend to be systematically affected by the prevalence of an outcome. This correspondence and responses to it are available through the Institutional Correspondence subpage of the Measuring Health Disparities page of jpscanlan.com. It includes letters to the Departments of Justice and Education explaining that, contrary to premises of federal civil rights enforcement policies, reducing adverse borrowing and discipline outcomes tend to increase, rather than reduce, relative differences in experiencing those outcomes. The responses to this correspondence are revealing of the limited ability of governmental institutions to comprehend and act on information of modest complexity even when such information indicates that the institutions’ understandings of crucial concepts are the exact opposite of reality.

Correspondence relating to Harvard University and certain related entities also warrant brief treatment here. It is possible, though by no means certain, that my 2009 and 2010 letters to the National Quality Forum and the Robert Wood Johnson Foundation had a role in causing those organizations to secure the services of Harvard Medical School and the Disparities Solution Center of Massachusetts General Hospital to produce a healthcare disparities measurement guide. The guide, *Commissioned Paper*: *Health Care Disparities Measurement* (Weissman et al. 2011), when released for public comment in the summer of 2011, was superior to many such guides in the scope of its coverage of measurement issues. It even pointed out, though somewhat obscurely, that it is possible for relative differences in favorable and adverse outcomes to yield different conclusions about directions of changes in disparities over time. But it showed no recognition whatever of the ways the measures it discussed tended to be affected by the prevalence of an outcome. That remained the case after the authors reviewed my comments bringing to their attention my work and the work of others, including NCHS, addressing such issues.^26^

By failing to address issues concerning the way measures tend to change simply because the prevalence of an outcome changes, the guide, in the final form issued in November 2011, will tend to lead readers to believe no such issues exist. It thus has far greater potential to undermine healthcare disparities research than to inform it.

In a lengthy October 2012 letter to Harvard University, written in conjunction with the applied statistics workshop referenced in note 2, and addressing a number of issues about health and health disparities research at Harvard Medical School and Harvard School of Public Health, I urged the University to take steps to have the *Commissioned Paper* withdrawn. In addition to summarizing the failings of the *Commissioned Paper*, the letter provides a fair summary of health and health disparities research at Harvard with respect to the measurement issues addressed in this article. It shows that none of that research recognizes that the measures on which it relies tend to be affected by the prevalence of an outcome. In fact, almost no Harvard research recognizes that a measure other than that employed in a study might or would yield a different conclusion concerning the comparative size of two disparities in outcome rates, and none of it would lead anyone to imagine that one might reach a different conclusion about directions of changes in disparities over time if one relied on relative differences in favorable outcomes rather than relative differences in adverse outcomes. Thus, as with research at other institutions around the world, both with respect to its main findings and its hypothesizing about the fact that one difference between outcome rates is larger than another, Harvard’s health and healthcare disparities research is almost invariably unsound.

Harvard did not respond to the letter. But the research integrity officers of Harvard Medical School and Massachusetts General Hospital did respond to a follow-up letter elaborating on the reasons why the *Commissioned Paper* should be withdrawn. The response stated that issues I raised concerning the guide involved “a difference of scientific opinion” and not research misconduct, and that, absent the latter, and two institutions do not independently assess the merits of individual papers of their faculty members. The institutions therefore declined to withdraw the guide.

The letter did not explain the perceived difference of opinion. But to the extent that there exists an articulable opinion contrary to my own concerning the *Commissioned Paper*, it would not merely be an opinion that one may in fact usefully explore such things as the reasons for increasing relative racial differences in some adverse outcome rate without consideration of the implications of the general declines in the outcome. It would also be an opinion that it is unnecessary for a measurement guide to alert readers that there exists a body of work maintaining that each of the measures discussed in the guide is fatally flawed unless employed with an understanding of the way it tends to change as the prevalence of an outcome changes.

The guide continues to bear on its cover the names of Harvard Medical School and Massachusetts General Hospital, as well as the National Quality Forum and the Robert Wood Johnson Foundation. Because of the stature of those entities, as well the guide’s currency, it is likely to influence a great deal of health and healthcare disparities research and, in doing so, contribute to the longstanding pattern whereby most of that research is wasteful even when it is not misleading. The guide also continues to serve as a foundation document for other health and healthcare disparities guidance of the National Quality Forum and presumably plays a significant role in the training of administrators in the Disparities Leadership Program of the Disparities Solution Center of Massachusetts General Hospital.

But it is a guide that should never have been published. And by continuing to support it the aforementioned institutions run the risk that they will undermine their credibility, not only with regard to the measurement of demographic differences, but with regard to the many more complex matters as to which to they are believed to have great expertise. Like issues exist with respect to health and healthcare disparities research at Harvard Medical School and Harvard School of Public Health, as well as Massachusetts General Hospital, if continued to be conducted without regard to the measurement issues that at least the research integrity officers of Harvard Medical School and Massachusetts General Hospital ought now to fully understand. Such officers should recognize as well the varied ethical issues in the conduct of research of any nature that cannot be fully defended, issues that are heightened when research is supported by government funds.

Many other institutions whose activities involve equally flawed analyses of demographic differences face similar risks. But few are in as good a position as Harvard and Massachusetts General Hospital to know better.

The ideas I expressed in “Race and Mortality” and varied other works about measuring demographic differences have yet to materially affect the way observers analyze demographic differences. But the issues I have raised are not matters of nuance. And whatever time it takes, I have little doubt that there will eventually be universal recognition of the patterns described here and of the implications of those patterns. The first such recognition may involve the research community’s appraisal of differences in health or healthcare outcomes or other social issues or that community’s interpretation of data in clinical contexts with respect to things like subgroup effects. Or it may involve the federal government’s either recognizing on its own, or being forced to recognize, that it encourages entities to take actions that make it more likely that the government will sue them. Once that recognition occurs in one of these areas, it may or may not spread quickly to the others. But it will eventually come in each of these areas as well as in others where the implications of the patterns have escaped my notice. It will be to the advantage of all that the recognition comes sooner rather than later.

## Footnotes

1 The 1.85 and 1.27 figures are usually termed “relative risks,” “risk ratios,” or “rate ratios” (RR). The relative difference is RR minus 1 where RR is greater than 1 (in which case the larger the RR the larger the relative difference) and 1 minus RR where RR is less than 1 (in which case the smaller the RR the larger the relative difference). I generally use the larger figure as the numerator of the RR for both favorable and adverse outcomes. Thus, as to both outcomes, the larger the RR the larger the relative difference. Whether one uses the larger or smaller figure as the numerator in RR can affect the size of a relative difference. For example, in a case were rates are 10 % and 20 %, the former could be deemed 50 % less than the latter (RR = 0.5) or the latter could be deemed 100 % greater than the former (RR = 2.0). But choice of numerator is irrelevant to issues about the comparative sizes of relative differences addressed here.

2 The principal pages are Measuring Health Disparities, Scanlan’s Rule, Mortality and Survival, Measuring Association, Immunization Disparities, Lending Disparities, Discipline Disparities, Educational Disparities, Disparate Impact, and Feminization of Poverty. As of the middle of 2014, these pages had something in excess of eighty subpages. Useful collections of graphical and tabular illustrations may be found in my October 2012 applied statistics workshop at Harvard’s Institute for Quantitative Social Science (“The Mismeasure of Group Differences in the Law and the Social and Medical Sciences”), my September 2013 paper for a University of Kansas School of Law faculty workshop (“The Mismeasure of Discrimination”), and my paper and presentation from the November 2013 Federal Committee on Statistical Methodology (FCSM) 2013 Research Conference (“Measuring Health and Healthcare Disparities”). I will refer to some of these illustrations later in this article.

3 These articles are listed on the Bibliography subpage of the Scanlan’s Rule page. The most significant was probably “The Perils of Provocative Statistics” in the Winter 1991 issue of *The Public Interest*. Like “Race and Mortality,” a number of the articles discussed the mistaken attention given to the so-called “feminization of poverty” by observers who failed to recognize that reductions in poverty (including the poverty of female-headed families) tended to increase the feminization of poverty, while increases in poverty (including the poverty of female-headed families) tended to reduce the feminization of poverty. That subject was also addressed in my article in the January/February 1992 issue of *Society* titled “The Curious Case of Affirmative Action for Women.”

4 These items and an internal NCHS effort to respond to “Race and Mortality” or other articulations of its principal points are discussed on the Consensus subpage of the Scanlan’s Rule page.

5 See slide titled “Healthy People 2010 Technical Appendix at A-18” in the FCSM presentation referenced in note 2.

6 In the NHDR, AHRQ attempts, consistent with the NCHS Healthy People guidance, to measure disparities in terms of relative difference in adverse outcomes even when the report describes the subject in terms of the favorable outcome. But it has not been invariably successful in doing so. In the 2012 report, among the situations highlighted as involving the most rapidly decreasing disparities are situations where relative differences in adverse outcomes in fact increased though relative differences in the corresponding favorable outcomes decreased. See Table 5 of the FCSM presentation and the NHDR Measurement subpage of the Measuring Health Disparities page.

7 See the AHRQ’s Vanderbilt Study subpage of the Measuring Health Disparities page.

8 See Table 13 of the FCSM presentation.

9 See discussion in the Immunization Disparities page of a study that examined both relative differences in favorable outcomes (with respect to receipt of full immunization) and relative differences in adverse outcomes (with respect to failure to receive any immunization), but without recognizing that general increases in immunization would tend to reduce the first relative difference while increasing the latter relative difference.

10 See the Mortality and Survival page and the Mortality/Survival Illustrations subpage of the Scanlan’s Rule page, as well as Table 12 of the FCSM presentation.

11 See Table 5 of the FCSM paper.

12 The handbook (at 172) recognized that the patterns of contrasting changes in the two relative differences were functions of the underlying distributions, a recognition not reflected in the NCHS documents.

13 Certain nuances of the pattern by which absolute differences tend to change as the prevalence of an outcome changes are discussed in the introductory section of the Scanlan’s Rule page.

14 The two relative differences and the absolute difference may all change in the same direction as the prevalence of an outcome changes, in which case one can infer that there occurred a true change in the strength of the forces causing the two groups’ rates to differ. But in the more common circumstances where all measures do not change in the same direction, the absolute difference will change in the same direction as the smaller relative difference.

15 The odds of selection for each group is the group’s selection rate divided by its rejection rate. As the prevalence of an outcome changes, the difference measured by the odds ratio tends to change in the opposite direction of the absolute difference. In order to lessen the complexity of this article I give the odds ratio only limited attention. But of the four standard measures discussed here, the odds ratio changes the least as the overall prevalence of an outcome changes and yields interpretations more closely approximating interpretations based on a theoretically sound measure than any of the other measures (as illustrated, for example, in Tables 3 and 4 of the Subgroup Effects subpage of the Scanlan’s Rule page).

16 After much thought on whether one could soundly measure health disparities, I developed this method in late 2007. But in 2010 I came to recognize that the method has been in existence since 1934 in what is known as the probit. See the Solutions subpage of the Measuring Health Disparities page.

17 Table 2 of the FCSM presentation shows for a variety of pairs of rates the EES and meaning of the EES in terms of the proportion of the disadvantaged group’s distribution that is above the mean of the advantaged group’s distribution.

18 For a fuller explanation of the problems with the described approach, see the Solutions, Irreducible Minimums, and Cohort Considerations subpages of the Measuring Health Disparities page and the Truncation Issues subpage of the Scanlan’s Rule page.

19 See the Pay for Performance and Between-Group Variance subpages of the Measuring Health Disparities page and pages 21 to 24 of the letter to Harvard University discussed infra.

20 Issues concerning the interpretation of subgroup effects, as well as issues pertaining to the manner in which a risk reduction observed in a clinical trial should be used to estimate the absolute risk reduction and number-needed-to-treat to avoid one adverse event in circumstances involving different baseline rates from that in the clinical trial, have great practical importance. But the treatment of such issues to date suffers from the same shortcomings as health disparities research. See the Subgroup Effects subpage of the Scanlan’s Rule page.

21 The United Kingdom has long been the leader in health disparities research and the Whitehall Studies have been a cornerstone of the research. But such research has never been any sounder in the UK than elsewhere. See my British Society for Population Studies 2006 Conference paper titled “The Misinterpretation of Health Inequalities in the United Kingdom.”

22 The Subgroup Effects subpage of the Scanlan’s Rule page shows how the EES can be used to determine whether the exists a subgroup effect in a clinical trial and provides a basis for employing an observed risk reduction in a clinical trial to estimate the absolute risk reduction for baseline rates different from those in the trial.

23 See the Whitehall Studies subpage of the Measuring Health Disparities page, the Disparities – High Income subpage of the Lending Disparities page and the Criminal Records Effects subpage of the Scanlan’s Rule page. For discussion of a complex issue concerning interpretations about the likelihood that racial differences in discipline rates result from teacher/administrator bias based on the fact that relative differences between black and white discipline rates are larger for subjectively-identified than objectively-identified misconduct, see the Offense Type Issues subpage of the Discipline Disparities page.

24 See the DOE Equity Report, California Disparities, Maryland Disparities, Los Angeles SWPBS, and Denver Disparities subpages of the Discipline Disparities page. See also the Suburban Disparities subpage, which discusses efforts to determine why relative racial difference in discipline rates are larger in Philadelphia’s suburbs than in the city itself without consideration of the implications of the generally low discipline rates in the suburbs.

25 See the Educational Disparities page and its subpages.

26 In response to my assertion that health and healthcare disparities research was universally flawed for failure to recognize the ways standard measures tend to be affected by the prevalence of an outcome, the authors added eight words at the end of a sentence in the report such that it would now read: “While calculations of disparities can be straightforward, comparisons of disparities among entities or over time can be sensitive to the calculations chosen, especially when the prevalence of an outcome changes.”
